# Procedure-related pain during CT-guided percutaneous transthoracic needle biopsies of lung lesions: a prospective study

**DOI:** 10.1186/s40644-023-00578-3

**Published:** 2023-06-12

**Authors:** Shou-Xin Yang, Mai-Lin Chen, Lei Xie, Hai-Bin Zhu, Yu-Liang Liu, Rui-Jia Sun, Bo Zhao, Xu-Bo Deng, Xiao-Ting Li, Ying-Shi Sun

**Affiliations:** 1 Key Laboratory of Carcinogenesis and Translational Research (Ministry of Education/Beijing), Department of Radiology, Peking University Cancer Hospital and Institute, No. 52, Fu Cheng Road, Hai Dian District, Beijing, 100142 China; 2 Department of Radiology, The First Affiliated Hospital of Shantou University Medical College, Shantou City, Guangdong Province 515041 China

**Keywords:** Lung, Image-guided biopsy, Pain

## Abstract

**Background:**

The existing data on the degree of pain in patients during CT-guided percutaneous transthoracic needle biopsy (PTNB) of lung lesions are limited and the factors related to pain are unclear. In this study, we aimed to evaluate the prevalence and severity of pain reported during PTNB and to identify factors associated with increased reported pain.

**Methods:**

Patients who underwent PTNB from April 2022 to November 2022 were prospectively evaluated using the numeric rating scale, which assesses subjective pain based on a 0–10 scoring system (0 = no pain; 10 = the worst pain imaginable). The scale divides the scores into three categories: mild pain (1–3 points), moderate pain (4–6 points), and severe pain (7–10 points). Pain scores from 4 to 10 were considered significant pain. Demographic data of patients, lesion characteristics, biopsy variables, complications, the patient’s subjective feelings, and pathological result data were analyzed by multivariable logistic regression analysis to identify variables associated with significant pain.

**Results:**

We enrolled 215 participants who underwent 215 biopsy procedures (mean age: 64.5 ± 9.3 years, 123 were men). The mean procedure-related pain score was 2 ± 2. Overall, 20% (43/215) of participants reported no pain (score of 0), 67.9% (146/215) reported pain scores of 1–3, 11.2% (24/215) reported scores of 4–6, and 0.9% (2/215) reported scores of 7 or higher. Furthermore, non-significant pain (scores of 0–3) was reported during 87.9% (189/215) of the procedures. In the adjusted model, significant pain was positively associated with lesions ≥ 34 mm (p = 0.001, odds ratio [OR] = 6.90; 95% confidence interval [CI]: 2.18, 21.85), a needle-pleural angle ≥ 77° (p = 0.047, OR = 2.44; 95% CI: 1.01, 5.89), and a procedure time ≥ 26.5 min (p = 0.031, OR = 3.11; 95% CI: 1.11, 8.73).

**Conclusions:**

Most participants reported no pain or mild pain from CT-guided percutaneous transthoracic needle biopsies of lung lesions. However, those with a larger lesion, a greater needle-pleural angle, and a longer procedure time reported greater pain.

**Supplementary Information:**

The online version contains supplementary material available at 10.1186/s40644-023-00578-3.

## Background

CT-guided percutaneous transthoracic needle biopsy (PTNB) is a widely established technique for obtaining histological diagnosis due to its high accuracy, ranging from 82 to 98% [1–5]. Moreover, PTNB is a safe and minimally invasive approach with a low severe complication rate. Some relatively common complications do occur, such as pneumothorax (in 15–42% of biopsies [5–9]) and hemorrhage (in 18–41% of biopsies [8–10]). However, procedure-related pain during the biopsy is a crucial yet under-investigated symptom.

The sensation of pain comes primarily from the stimulation of nerves, and the pain from sharp instruments and the fear of unknown pain can be extremely uncomfortable for the patient. Procedure-related pain is critical for the patient’s experience and the success of the biopsy, as pain can cause undesirable patient motion [11]. However, few studies have explored the pain associated with percutaneous biopsies of lung lesions [12, 13]. In the era of precision medicine and as the need for PTNBs of lung lesions increases [14], radiologists play a more considerable role in direct patient care. Thus, understanding the prevalence and influencing factors of procedure-related pain can help radiologists appropriately counsel patients before their procedure and help operators know how and when to anticipate and manage pain [15].

During a PTNB of lung lesion, the most sensitive structures in the needle’s path are the skin and parietal pleural; pain mainly arises when they are pierced [16, 17]. Consequently, the consensus is that pleural anesthesia is essential in addition to skin anesthesia [18], supported by a previous study that demonstrated pleural anesthesia helps reduce pain [19]. However, few studies have assessed a patient’s subjective pain during a lung biopsy after administering skin and pleural anesthesia. Additionally, limited data exist regarding factors associated with increased pain perception. We hypothesized that some factors would influence the procedure-related pain during biopsies. Therefore, this study aimed to assess subjective pain levels during PTNB of pulmonary lesions and analyze factors associated with greater pain scores.

## Materials and methods

### Study participants

Our hospital’s institutional review board approved this study, and all participants provided written informed consent. Consecutive patients who underwent a PTNB of pulmonary lesions from April 2022 to November 2022 were prospectively included. Patients with subjective pain before the biopsy were excluded. We interviewed patients immediately after the biopsy using a questionnaire that included the numeric rating scale for pain [20].

### Biopsy procedure

All PTNBs were performed under CT guidance on a 64-detector row CT scanner (Optima CT 680; GE Healthcare, Chicago, IL, USA); 2.5-mm or 1.25-mm helical images were obtained. The radiologist (M.C., 10 years of PTNB experience) performed all biopsies using a semi-automatic cutting biopsy needle (18-gauge; Bard® Magnum® Biopsy Instrument; BD, Franklin Lakes, NJ, USA) and the coaxial technique.

All participants underwent simultaneous contrast-enhanced CT examination in our hospital within three weeks before the biopsy. The operator thoroughly reviewed the contrast-enhanced CT images before the biopsy to determine the optimal needle path and appropriate patient position (supine or prone). After the participant took the appropriate position, the CT exam was performed. The expected path from the skin to the target lesion was drawn with a line on the console monitor to determine the angle and distance for the needle path.

**Fig. 1:**
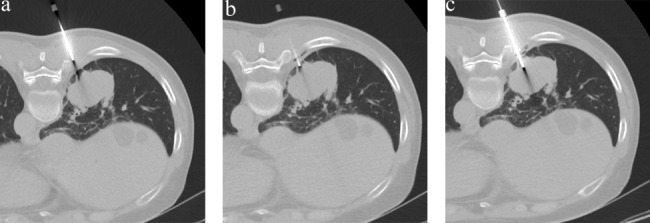
Axial CT images of a 56-year-old woman with a mass in the right lower lobe, who received pleural anesthesia. **a**. The coaxial needle entered the extra-thoracic muscle according to the given direction and angle. **b**. After injecting a small amount of anesthetic, CT was taken to confirm the position. Accumulation of anesthetic and incidental air bubbles is noted in the extra-pleural space. **c**. The coaxial introducer passed through the pleural and was further advanced to place the tip in the target lesion. The patient complained of mild pain (pain score 2).

All participants received skin and pleural anesthesia. After skin sterilization and orally informing the participant about the procedure, skin anesthesia was administered by a subepidermal injection of a small quantity of 1% lidocaine solution to create a blister. The distance from the skin to the pleura was measured on the line for the needle path. Then, a 17-gauge coaxial introducer was inserted into the chest wall along the pre-drawn line and angle for the needle path. The introducer was advanced until the operator positioned the needle tip in the extra-pleural space between the endothoracic fascia and parietal pleura. Then, the stylet was removed from the introducer, and 2–3 mL of anesthetic was injected to anesthetize the parietal pleura (Fig. 1) as previously described [18]. The stylet was reinserted into the introducer, and the introducer was inserted into the target lesion under intermittent CT guidance.

The stylet was removed after confirming that the needle tip was in the lesion. Sampling was performed by passing an 18-gauge semi-automated cutting needle. One to four samples were acquired, and the operator visually assessed whether the sampling was sufficient. Immediately after removing the coaxial introducer, a CT scan was performed to identify procedure-related complications.

### Data collection and analysis

Pain was assessed by another radiologist (S.Y., 2 years of PTNB experience) immediately after the biopsy using the numeric rating scale and three related questions: (1) In your past experience, were you a person who was particularly afraid of pain? (2) Was the body positioning during the biopsy uncomfortable? and (3) Did you have any symptoms other than pain? The patients were asked to assign a number to their pain level on a scale of 0–10 (0 = no pain; 10 = the worst pain imaginable). The scale divides the scores into three categories: mild pain (1–3 points), moderate pain (4–6 points), and severe pain (7–10 points) [21].

Data on potential factors influencing pain were also collected, including age, sex, lesion size, lesion type (solid, part-solid), lesion location, pleural traction, emphysema along the needle path, patient position (prone, supine), the needle-pleural angle (0–90), traversal of a fissure, lesion depth (distance from the pleura to the lesion along the needle path), number of scans, procedure time, and radiation dose. Complications including pneumothorax, pulmonary hemorrhage, and air embolism, were also recorded. The final pathological diagnosis was classified as diagnostic (specific benign or malignant results) or non-diagnostic (no specific diagnosis or insufficient organization for diagnosis).

### Statistical methods

Categorical variables are presented as numbers (percentage), and continuous variables are presented as means (standard deviations) or medians and interquartile ranges (25th − 75th percentiles), as appropriate. Pain scores were dichotomized into non-significant (scores ≤ 3) and significant (scores ≥ 4) pain. The continuous variables, lesion size, needle-pleural angle, and procedure time were dichotomized based on an optimal cut-off determined by receiver operating characteristic curves applying Youden’s index. Independent sample t-test, Mann–Whitney U test, Pearson’s chi-square test, and Fisher’s exact test were used to compare differences across groups, as appropriate. Factors associated with significant pain were identified by multivariable logistic regression analyses. Variables with a p-value of < 0.10 in the univariable analyses were used in the multivariable analysis. P-values of < 0.05 (two-tailed testing) were considered significant. All analyses were performed using SPSS version 25.0 for Windows (IBM Corp., Armonk, NY, USA).

## Results

### Patient demographics

**Fig. 2:**
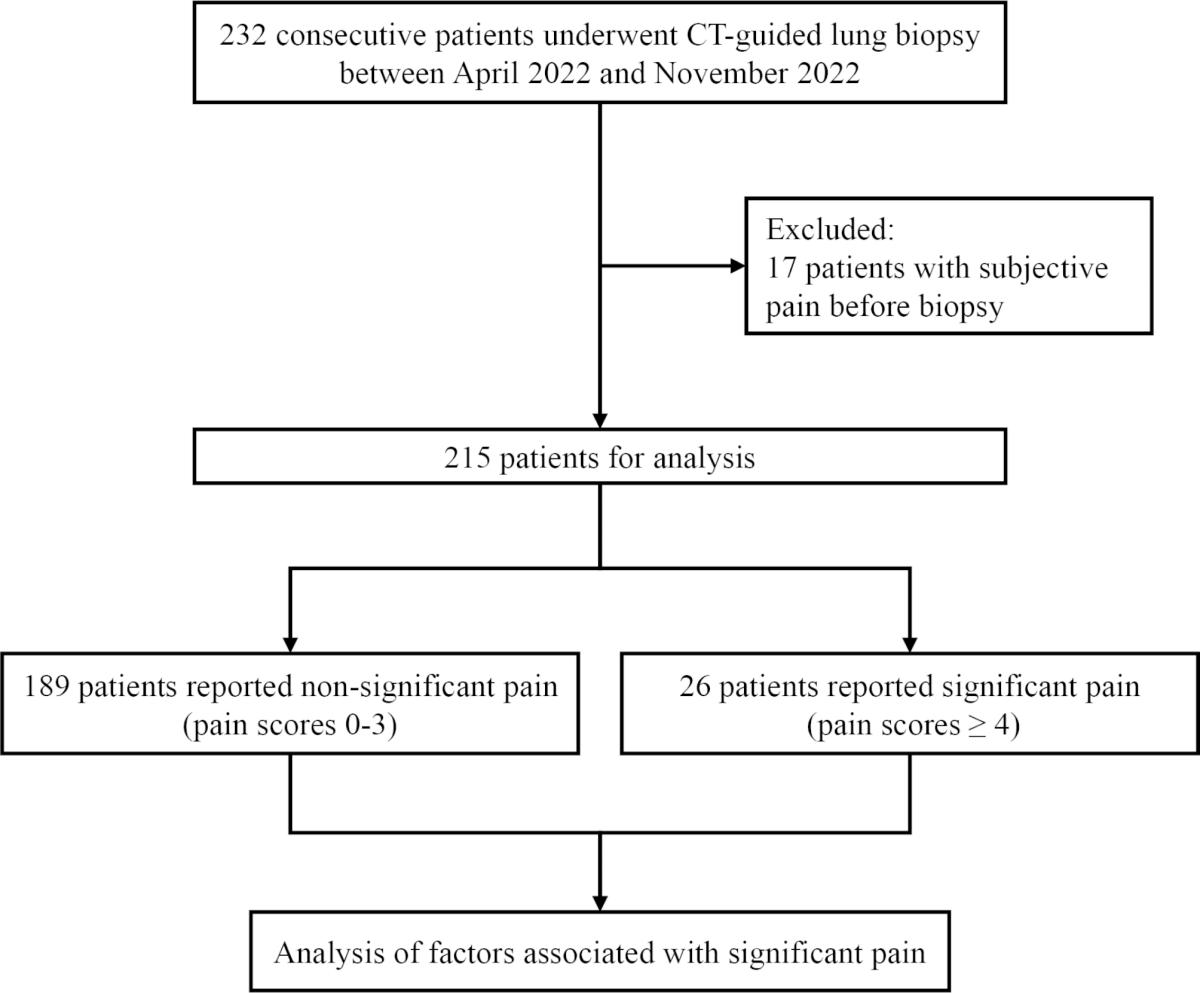
Flowchart shows inclusion and exclusion criteria for the study

Between April 2022 and November 2022, 232 consecutive patients met the inclusion criteria. However, 17 patients with subjective pain before the biopsy were excluded. Thus, the analysis set included 215 biopsies in 215 participants (mean age, 64.5 ± 9.3 years; range, 29–85 years; Fig. 2). The cohort included 123 (57.2%) men (mean age, 65.4 ± 9.1 years) and 92 (42.8%) women (mean age, 63.2 ± 9.6 years) (Table 1). The mean lesion size was 38.1 ± 18.6 mm, the mean needle-pleural angle was 68.6 ± 14.6°, and the median procedure time was 18 mins (interquartile range = 14–23).

**Table 1:** Univariate analysis to determine distinguishing variables of significant pain from non-significant pain

Characteristics	All(n = 215)	Non-significant pain (n = 189)	Significant pain (n = 26)	*p*-value
Age(years)*	64.5 ± 9.3	64.5 ± 9.5	64.5 ± 8.1	0.997
Sex, female	92(42.8)	81(42.9)	11(42.3)	0.958
Lesion size ≥ 34 mm	116(54.0)	94(49.7)	22(84.6)	**0.001**
Lesion type				
Solid	193(89.8)	172(91.0)	21(80.8)	0.157
Part-solid	22(10.2)	17(9.0)	5(19.2)	
Lesion location				
Left upper lobe	55(25.6)	46(24.3)	9(32.6)	0.445
Left lower lobe	36(16.7)	34(18.0)	2(7.7)	
Right upper lobe	69(32.1)	62(32.8)	7(26.9)	
Right middle lobe	10(4.7)	8(4.2)	2(7.7)	
Right lower lobe	45(20.9)	39(20.6)	6(23.1)	
Pleural traction, yes	104(48.4)	92(48.7)	12(46.2)	0.809
Emphysema along the needle path	50(23.3)	44(23.3)	6(23.1)	0.982
Position				
Prone	122(56.7)	111(58.7)	11(42.3)	0.113
Supine	93(43.3)	78(41.3)	15(57.7)	
Needle-pleural angle (≥ 77°)	72(33.5)	58(30.7)	14(53.8)	**0.019**
Traversal of fissure	8(3.7)	7(3.7)	1(3.8)	1.000
Lesion depth (mm)*	24.7 ± 16.1	24.8 ± 16.3	23.8 ± 15.0	0.760
Number of scans†	9(7, 12)	9(7, 12)	9(7,12.25)	0.968
Procedure time ≥ 26.5 mins †	36(16.7)	28(14.8)	8(30.8)	**0.051**
Radiation dose (DLP, mGy cm^2^) †	684.5(436.1,891.9)	679.0(426.2, 884.5)	759.2(558.4,1036.7)	0.158
Hemorrhage	168(78.1)	147(77.8)	21(80.8)	0.729
Pneumothorax	88(40.9)	81(42.9)	7(26.9)	0.121
Particular fear of pain	48(22.3)	40(21.2)	8(30.8)	0.270
Uncomfortable about the position	17(7.9)	13(6.9)	4(15.4)	0.133
Diagnostic results	194(90.2)	169(89.4)	25(96.2)	0.464

Variables used as inputs to multivariate analysis are indicated in bold. DLP, dose-length product

*Data are mean ± standard deviation, †Data are median and interquartile range (IQR, 25th -75th percentile)

### Pain scores, complications and histological outcomes

**Fig. 3:**
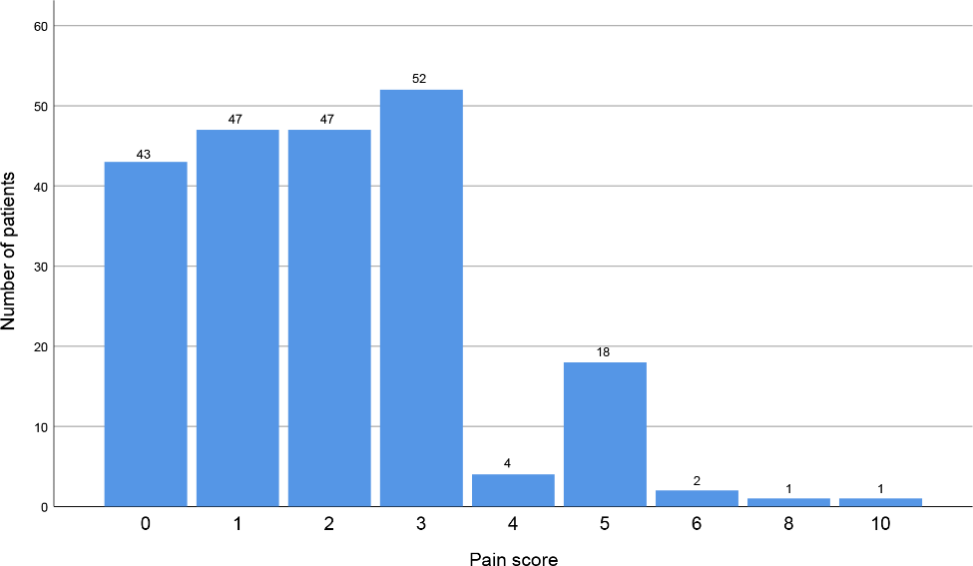
Chart shows distribution of pain scores patient reported during biopsy. Patients scored pain on 0–10 numeric rating scale (0, no pain; 10, worst pain imaginable).

**Fig. 4:**
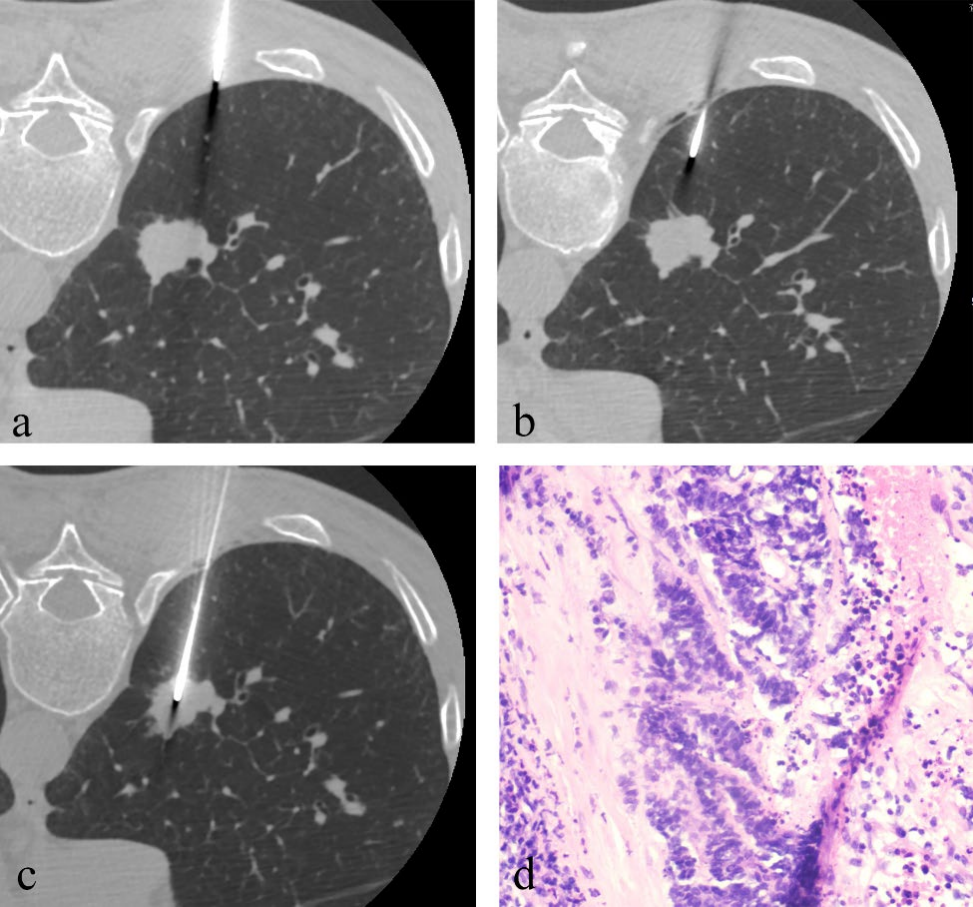
Axial CT images of a 61-year-old man with a 23-mm mass in the right lower lobe. (**a**-**c**). The coaxial introducer was passed through the chest wall and pleura step by step to reach the inside of the lesion. Needle-pleura angle was 45° and the procedure time was 15 min. Patient reported a pain score of 3. (**d**). The photomicrograph showed that the pathological result was small cell carcinoma (Hematoxylin-eosin stain, ×100).

**Fig. 5:**
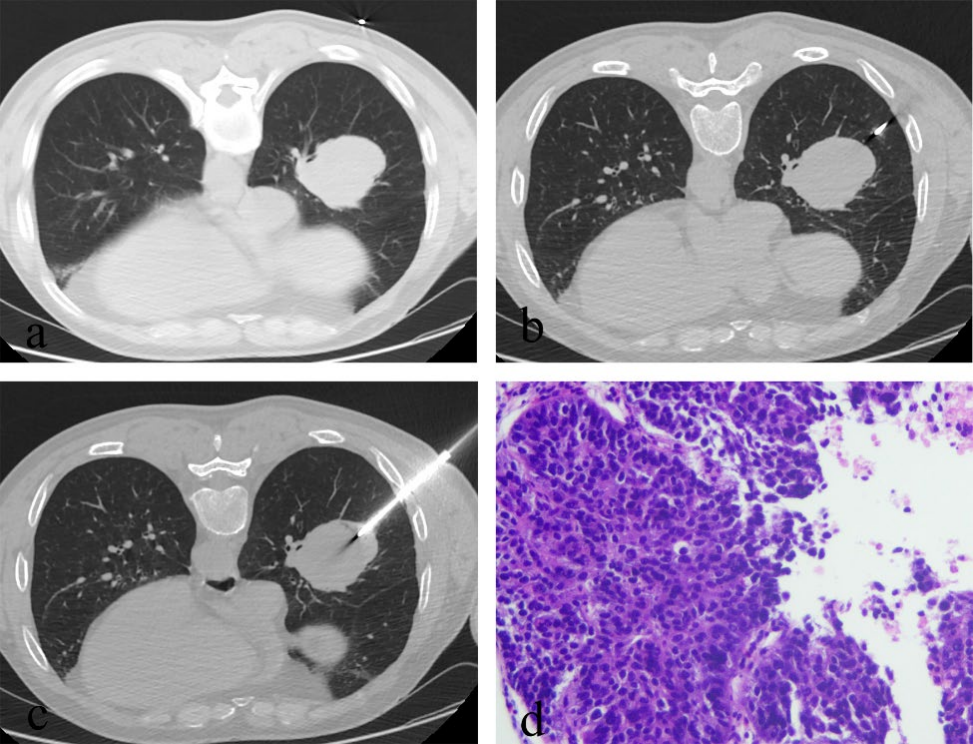
Axial CT images of a 62-year-old man with a 60-mm mass in the right lower lobe. (**a**-**c**). The coaxial introducer was passed through the chest wall and pleura step by step to reach the inside of the lesion. Needle-pleura angle was 80° and the procedure time was 16 mins. Patient reported a pain score of 5. (**d**). The photomicrograph showed that the pathological result was squamous cell carcinoma (Hematoxylin-eosin stain, ×100).

The overall mean procedure-related pain score was 2 ± 2. Figure 3 details the pain distributions; 20% (43/215) of patients reported no pain, 67.9% (146/215) reported a score of 1–3, 11.2% (24/215) reported a score of 4–6, and 0.9% (2/215) reported a score of 7 or higher. Figures 4 and 5 show the pain scores of two patients. Since only two participants reported pain scores above 7 (specifically, scores of 8 and 10), we conducted telephone follow-ups for these two patients within two weeks. The participants with pain scores of 8 and 10 said they still felt pain at the time of telephone follow-up.

Regarding the questionnaire, 22.3% (48/215) of the participants reported being particularly afraid of pain, 7.9% (17/215) reported feeling uncomfortable about their body position during the biopsy, and 7.4% (16/216) had symptoms other than pain, primarily chest discomfort (7/16) and cough (5/16). However, other symptoms included nausea (1/16), fatigue (1/16), lumbar discomfort (1/16), and hemoptysis (1/16).

The overall incidence of pneumothorax, pulmonary hemorrhage, hemothorax, and air embolism was 40.9% (88/215), 78.1% (168/215), 0.9% (2/215) and 0.9% (2/215), respectively. Thoracic drainage was required for 1.4% (3/215) of the patients. Overall, 90.2% (194/215) of the biopsies led to diagnostic results.

### Non-significant pain (pain scores ≤ 3) versus significant pain (pain scores ≥ 4): factors associated with significant pain

Overall, 87.9% (189/215) reported non-significant pain and 12.1% (26/215) reported significant pain. The receiver operating characteristic curves determined that the optimal cut-off points were: lesion size: 34 mm, needle-pleural angle: 77°, and procedure time: 26.5 mins (Supplementary Table 1). The participants were divided into two groups based on these cut-off points.

Table 1 summarizes the participants’ demographic data based on non-significant and significant pain. Multiple logistic regression included lesion size (≥ 34 mm), the needle-pleural angle (≥ 77°), and the procedure time (≥ 26.5 mins) based on the univariable analysis. In the adjusted model, significant pain was positively associated with a lesion size ≥ 34 mm (p = 0.001, OR = 6.90, 95% CI: 2.18, 21.85), the needle-pleural angle ≥ 77° (p = 0.047, OR = 2.44, 95% CI: 1.01, 5.89), and the procedure time ≥ 26.5 mins (p = 0.031, OR = 3.11, 95% CI: 1.11, 8.73) (Table 2).

**Table 2:** Results of multivariate logistic regression analysis to for predicting significant pain

Variables	Adjusted OR	95% CI	p-value
Lesion size ≥ 34 mm	6.90	2.18–21.85	**0.001**
Needle-pleural angle ≥ 77°	2.44	1.01–5.89	**0.047**
Procedure time ≥ 26.5 mins	3.11	1.11–8.73	**0.031**

Statistically significant p values are indicated in bold. OR odds ratio, CI confidence interval

## Discussion

In this study, 87.9% of the participants reported non-significant pain during the biopsy procedure. However, those with a lesion ≥ 34 mm, a needle-pleural angle ≥ 77°, and a procedure time ≥ 26.5 mins were more likely to report significant pain. Conversely, the patient’s position, discomfort about their position, and particular fear of pain were not associated with significant pain.

Our results were similar to those of previous studies that reported no or mild pain during lung biopsies in 65.8–100% of patients [12, 13, 19, 22]. These results are reassuring because they indicate that most patients are comfortable with the biopsy. We also found that only 0.9% (2/215) of the participants had severe pain. Therefore, these data may help ease patient fears during the preprocedural evaluation.

The data on procedure-related pain are primarily limited to percutaneous parenchymal liver biopsies [23–27]. However, procedure-related pain during lung lesion biopsies has not been widely investigated [12, 13, 19, 22] nor has it received enough attention in daily practice, although patients are often anxious about procedure-related pain. We found that 80.0% (172/215) of the participants sensed pain, but only 12.1% (26/215) of patients reported significant pain during the biopsy, which is lower than that in a recently published study where 34.2% of patients reported significant pain [19]. This difference may be because some of their patients did not receive pleural anesthesia. Also, all biopsies in our study were conducted by the same experienced radiologist; thus, we controlled for operator differences.

In our study, a longer procedure time was associated with higher pain scores, perhaps because as the procedure time increases, the chance of irritating the pleura increases [19]. However, we also suspect that the strength of the anesthetic weakens with longer procedure times. Significant pain is also positively associated with large lesion size and needle-pleural angle. The possible reason is that for larger lesions, the operator will generally choose a longer penetration depth under safe conditions, and the longer penetration depth and greater cutting impact may cause pain to the patient. Furthermore, a larger needle-pleural angle means the needle is inserted perpendicular to the pleura, potentially applying more pressure during penetration, causing severe pain. The factors leading to significant pain identified in our study can be easily assessed in clinical practice; thus, operators could easily predict and manage a patient’s pain.

The current results validate those of a prior study that reported no relationship between the patients’ position and pain [19]. In addition, the questionnaire data helped confirm that the discomfort caused by the patient’s position and procedure-related pain are not associated, similar to a previous study that reported body position discomfort was primarily due to shoulder and neck stiffness [12]. We also found that patient’s sensitivity to pain was not associated with procedure-related pain, which should help appease pain-sensitive patients before an operation. A previous study reported that younger patients experienced more pain during a lung biopsy, possibly owing to less exposure to medical interventions and a lack of comprehension of the biopsy [13]. Our study did not confirm this, perhaps because almost all patients were fully informed of the biopsy process and precautions before surgery, and the number of participants in our study was relatively small.

Only two patients had severe pain, but the pain still existed to varying degrees at the two-week follow-up. Therefore, extra attention and follow-up consultations should be provided to patients with severe procedure-related pain. Moreover, we did not identify the factors associated with severe pain in the two cases, and further studies may be needed to explore the potential factors associated with the pain. Our incidence of pneumothorax was similar to that in prior reports, ranging from 15 to 42% [5–9]. Our incidence of pulmonary hemorrhage (78.1%) was higher than previously reported values [8–10], but only 0.9% (2/215) of patients had a small amount of hemothorax.

This study has several limitations. First, it is a single-center study with relatively few participants. Future studies should incorporate larger sample sizes. Second, this study did not evaluate some factors, such as the operators’ experience, biopsy needle type, the patient’s education level, preoperative anxiety, and expected pain, which may influence pain perception. These potential influencing factors should be fully considered in subsequent studies.

## Conclusions

PTNB of lung lesions is relatively comfortable, and most patients report non-significant pain. However, participants with larger lesions, larger needle-pleural angles, and longer procedure times reported more severe pain. This information may assist with preprocedural counseling and thus reassure patients before the procedure.

## Electronic supplementary material

Below is the link to the electronic supplementary material.

Supplementary file: Table S1

## Data Availability

Not applicable.

## Abbreviations

CT: -guided percutaneous transthoracic needle biopsy (PTNB)

## References

[1] Schreiber G, McCrory DC (2003). Performance characteristics of different modalities for diagnosis of suspected lung cancer: summary of published evidence. Chest.

[2] Tsukada H, Satou T, Iwashima A (2000). Diagnostic accuracy of CT-guided automated needle biopsy of lung nodules. Am J Roentgenol.

[3] Geraghty PR, Kee ST, McFarlane G (2003). CT-guided transthoracic needle aspiration biopsy of pulmonary nodules: needle size and pneumothorax rate. Radiology.

[4] Priola AM, Priola SM, Cataldi A (2007). Accuracy of CT-guided transthoracic needle biopsy of lung lesions: factors affecting diagnostic yield. Radiol Med.

[5] Choi JW, Park CM, Goo JM (2012). C-arm cone-beam CT-guided percutaneous transthoracic needle biopsy of small (≤ 20 mm) lung nodules: diagnostic accuracy and complications in 161 patients. Am J Roentgenol.

[6] Kuban JD, Tam AL, Huang SY (2015). The Effect of Needle Gauge on the risk of pneumothorax and chest tube Placement after Percutaneous computed Tomographic (CT)-Guided lung biopsy. Cardiovasc Inter Rad.

[7] Hiraki T, Mimura H, Gobara H (2010). Incidence of and risk factors for pneumothorax and chest tube placement after CT fluoroscopy-guided percutaneous lung biopsy: retrospective analysis of the procedures conducted over a 9-year period. Am J Roentgenol.

[8] Li Y, Du Y, Yang HF (2013). CT-guided percutaneous core needle biopsy for small (≤ 20 mm) pulmonary lesions. Clin Radiol.

[9] Heerink WJ, de Bock GH, de Jonge GJ (2017). Complication rates of CT-guided transthoracic lung biopsy: meta-analysis. Eur Radiol.

[10] Tai R, Dunne RM, Trotman-Dickenson B (2016). Frequency and severity of pulmonary hemorrhage in patients undergoing percutaneous CT-guided transthoracic lung biopsy: Single-Institution experience of 1175 cases. Radiology.

[11] Patatas K, Koukkoulli A (2009). The use of sedation in the radiology department. Clin Radiol.

[12] Wong ET, Dunham C, Patsios D (2014). Qualitative assessment of pain management in patients undergoing computed tomography-guided transthoracic lung biopsy. Pain Res Manag.

[13] Iyer VR, Sheedy SP, Gunderson TM (2019). Procedure-Related Pain during Image-Guided percutaneous biopsies: a retrospective study of prevalence and predictive factors. Am J Roentgenol.

[14] Mazzone PJ, Lam L (2022). Evaluating the patient with a pulmonary nodule: a review. Jama-J Am Med Assoc.

[15] Mueller PR, Biswal S, Halpern EF (2000). Interventional radiologic procedures: patient anxiety, perception of pain, understanding of procedure, and satisfaction with medication–a prospective study. Radiology.

[16] Brims FJ, Davies HE, Lee YC (2010). Respiratory chest pain: diagnosis and treatment. Med Clin N Am.

[17] Charalampidis C, Youroukou A, Lazaridis G (2015). Pleura space anatomy. J Thorac Dis.

[18] Gorgos AB, Ferraro P, Chalaoui J (2015). Percutaneous CT-guided lung interventions-local pleural anesthesia. Clin Imag.

[19] Beck KS, Chang S, Han DH (2021). The effectiveness and safety of local pleural anesthesia for pain control in patients undergoing CT-guided transthoracic needle biopsy. Eur Radiol.

[20] Karcioglu O, Topacoglu H, Dikme O (2018). A systematic review of the pain scales in adults: which to use?. Am J Emerg Med.

[21] Serlin RC, Mendoza TR, Nakamura Y (1995). When is cancer pain mild, moderate or severe? Grading pain severity by its interference with function. Pain (Amsterdam).

[22] Winn N, Spratt J, Wright E (2014). Patient reported experiences of CT guided lung biopsy: a prospective cohort study. Multidiscip Resp Med.

[23] Akay S, Karasu Z, Noyan A et al. Liver biopsy: is the pain for real or is it only the fear of it? Acute pain: international journal of acute pain management. 2007;9:95–6.

[24] Cadranel JF, Rufat P, Degos F (2000). Practices of liver biopsy in France: results of a prospective nationwide survey. For the Group of Epidemiology of the French Association for the study of the liver (AFEF). Hepatology.

[25] Eisenberg E, Konopniki M, Veitsman E (2003). Prevalence and characteristics of pain induced by percutaneous liver biopsy. Anesth Analg.

[26] Castera L, Negre I, Samii K (1999). Pain experienced during percutaneous liver biopsy. Hepatology.

[27] Chevallier P, Ruitort F, Denys A (2004). Influence of operator experience on performance of ultrasound-guided percutaneous liver biopsy. Eur Radiol.

