# Supplementary material for: Procedure-related pain during CT-guided percutaneous transthoracic needle biopsies of lung lesions: a prospective study
Source: Cancer Imaging. 2023 Jun 12;23:61. doi: 10.1186/s40644-023-00578-3 (PMC10259012; doi:10.1186/s40644-023-00578-3)
Supplement: Supplementary file 1 — Supplementary file: Table S1 [file 40644_2023_578_MOESM1_ESM.docx]

Supplementary Table 1 The continuous variables, lesion size, needle-pleural angle, and procedure time were dichotomized around an optimal cut-off identified on ROC curves applying Youden’s index

|  | Cut off | AUC | AUC 95% CI | |
| --- | --- | --- | --- | --- |
| Lesion size, mm | 34 | 0.674 | 0.575 | 0.773 |
| Needle-pleural angle, ° | 77 | 0.616 | 0.497 | 0.734 |
| Procedure time, minutes | 26.5 | 0.580 | 0.455 | 0.704 |

AUC, area under the curve; CI, confidence interval; ROC, receiver operating characteristic.
